# The Incidence of Abemaciclib-induced Interstitial Lung Disease: A Single-center Retrospective Study in Japan

**DOI:** 10.24546/0100497175

**Published:** 2025-08-07

**Authors:** TAKESHI HASHIMOTO, HARUNA NAKAMURA, YOKO SAKODA, KAZUHIKO TSUCHIYA, MAO FUJII, MASATO TAKI, SHUNTARO TOKUNAGA, SUYA HORI, TERUAKI NISHIUMA, MITSUTOSHI OGINO

**Affiliations:** 1 Department of Breast Surgery, Kakogawa Central City Hospital, Kakogawa, Japan; 2 Division of Breast and Endocrine Surgery, Kobe University Graduate School of Medicine, Kobe, Japan; 3 Department of Respiratory Medicine, Kakogawa Central City Hospital, Kakogawa, Japan

**Keywords:** Breast cancer, Interstitial lung disease, ILD, Abemaciclib, CDK4/6 inhibitor

## Abstract

**BACKGROUND AND AIM:**

Abemaciclib, a CDK4/6 inhibitor, is used for estrogen receptor (ER)-positive, human epidermal growth factor receptor 2 (HER2)-negative breast cancer. Interstitial lung disease (ILD) is a frequent adverse event of abemaciclib, particularly in Asian patients, though limited information is available on its incidence and risk factors. This study aimed to identify the incidence and risk factors of abemaciclib-induced ILD through a single-center retrospective analysis.

**METHODS:**

We analyzed ER-positive, HER2-negative inoperable or metastatic breast cancer patients treated with abemaciclib at Kakogawa Central City Hospital between November 1, 2018, and March 31, 2022. At least two respiratory medicine specialists evaluated computed tomography and examined the development of ILD after the initiation of abemaciclib. We conducted univariate analysis to examine factors associated with the development of ILD.

**RESULTS:**

Forty-nine patients were analyzed. The median (range) observation period was 27.0 (10–49) months, and the median (range) duration of abemaciclib was 11.0 (1–43) months. Fourteen patients (28.6%) received abemaciclib as a 3rd-line treatment or later. Ten patients (20.4%) were diagnosed with abemaciclib-induced ILD; 7 were diagnosed within 6 months of the initiation of abemaciclib and 3 developed severe ILD during the same period. We identified lung metastasis as a risk factor for the development of ILD (odds ratio = 5.00, 95% confidence interval: 1.15–21.70; p = 0.032).

**CONCLUSION:**

The incidence of abemaciclib-induced ILD was 20.4%, which was higher than previously reported values. Today, as abemaciclib is one of the standard treatments for ER-positive, HER2-negative breast cancer, we should be more careful about ILD.

## INTRODUCTION

Breast cancer is currently the most commonly diagnosed cancer among women. Approximately 2.3 million women worldwide were newly diagnosed with invasive breast cancer in 2020 (1). Breast cancer is mainly classified into four subtypes based on the expression of the estrogen receptor (ER) and human epidermal growth factor receptor 2 (HER2) (2). The treatment strategy is modified according to these subtypes. Approximately 70% of patients have ER-positive, HER2-negative breast cancer (3), and the combination of endocrine therapy and a cyclin-dependent kinase 4 and 6 (CDK4/6) inhibitor is one of the standard treatments for this subtype (4–7). CDK4/6 inhibitors suppress CDK4/6 expression and the phosphorylation of retinoblastoma, thereby arresting the cell cycle and inhibiting cell proliferation (8).

Abemaciclib is a CDK4/6 inhibitor that has been used to treat ER-positive, HER2-negative metastatic breast cancer patients since 2018 in Japan. Diarrhea and nausea are frequently reported as adverse events (AEs) of abemaciclib (4–7) and are manageable with antidiarrheals and antiemetics. However, one of the specific and uncontrollable AEs of abemaciclib is interstitial lung disease (ILD), which is a risk factor for death in Japan and other countries (9). The incidence of abemaciclib-induced ILD in Western countries was previously reported to be 2.3–3.4% (4–7), while that in Japanese patients was 13% (10).

Since the incidence of abemaciclib-induced ILD was higher in patients treated at Kakogawa Central City Hospital than in previous studies (3–6, 9), we performed a single-center retrospective analysis to identify risk factors and for a careful follow-up.

## MATERIALS AND METHODS

### Patients

Patients with ER-positive, HER2-negative inoperable or metastatic breast cancer treated with abemaciclib at the Breast Surgery Department of Kakogawa Central City Hospital between November 1, 2018, and March 31, 2022, were enrolled. All patients received abemaciclib for at least 28 days, and patients with a previous history of abemaciclib were excluded. In the present study, there were no restrictions on the treatment lines of abemaciclib.

### Methods

We collected the following information on these patients at the initiation of abemaciclib: age, sex, metastatic sites of breast cancer, previous treatments, combined endocrine therapy, the number of treatment lines, history of radiation therapy to the chest, and smoking history. After the initiation of abemaciclib, computed tomography (CT) of the chest and abdomen was performed once every 3 months with the consent of patients. CT was also conducted when disease progression such as new metastases was suspected, or symptoms including fever or cough developed.

Two respiratory medicine specialists were assigned to each case to evaluate CT images independently in this study. The baseline pulmonary condition was assessed based on CT before the initiation of abemaciclib. We examined the development of ILD after the initiation of abemaciclib. In patients diagnosed with ILD, we measured the time from the initiation of abemaciclib to the diagnosis of ILD, the shading patterns, and the severity of ILD based on the grades of Common Terminology Criteria for Adverse Events (CTCAE) v5.0 (11). When the CT evaluation by two specialists differed, a third specialist investigated whether ILD had developed and its severity. We calculated the number and percentage of patients who developed abemaciclib-induced ILD. Possible risk factors for the development of ILD were then analyzed. We performed statistical analyses using EZR (Saitama Medical Center, Jichi Medical University, Japan), a graphical user interface of R (The R Foundation for Statistical Computing, Vienna, Austria) (12). More precisely, EZR is a modified version of R commander designed to add statistical functions frequently used in biostatistics. We performed univariate logistic regression analysis to examine the association between ILD development and various clinical factors, including patient characteristics, comorbidities, metastatic sites, and prior treatments.

This study was approved by the Ethics Committee of Kakogawa Central City Hospital. Informed consent was obtained in the form of opt-out.

## RESULTS

**Figure 1:**
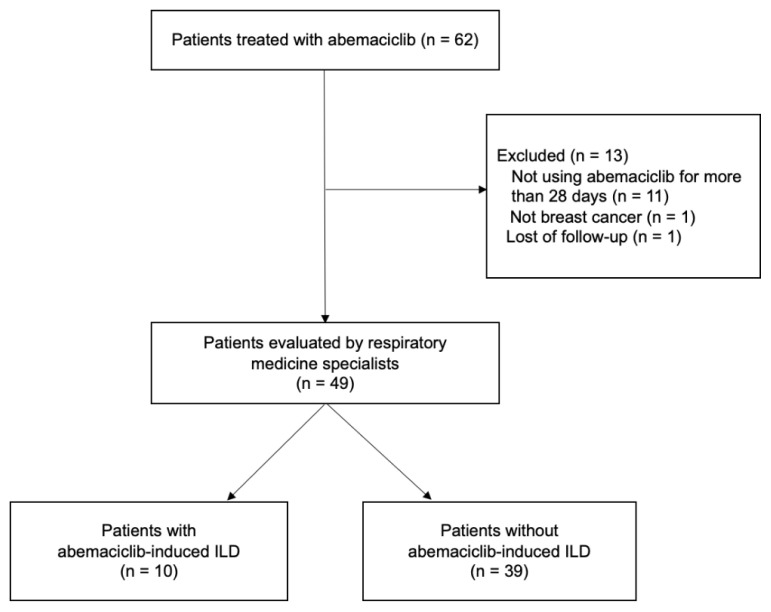
Patient flow diagram

Sixty-two patients were treated with abemaciclib between November 1, 2018, and March 31, 2022. Thirteen patients were excluded: 11 did not use abemaciclib for more than 28 days, 1 did not have metastatic breast cancer, and 1 was lost to the follow-up. Therefore, 49 patients were ultimately evaluated (Figure 1). We assessed the incidence of ILD until February 6, 2023. The median (range) observation period was 27.0 (10–49) months, and the median (range) duration of abemaciclib was 11.0 (1–43) months.

Table I shows patient characteristics. All patients were female and were diagnosed with ER-positive breast cancer. Twenty-one patients (42.9%) had a history of radiation therapy to the chest. None of the patients analyzed had interstitial pneumonia at the time of the initiation of abemaciclib.

Table II shows clinical characteristics. The most common endocrine therapy combined with abemaciclib was fulvestrant (35 patients, 71.4%), followed by aromatase inhibitors (13 patients, 26.5%). Bone was the most common site of metastasis, detected in 22 patients (44.9%). Abemaciclib was administered to 14 patients (28.6%) as a 3rd-line treatment or later. At the end of the observation period, 14 patients (28.6%) were still receiving abemaciclib. The number of patients with prior treatment with palbociclib or everolimus was 6 (12.2%) and 1 (2.0%), respectively. Thirty-five patients (71.4%) had discontinued abemaciclib: 18 (36.7%) due to progressive disease (PD) and 17 (34.7%) due to AEs. Abemaciclib was discontinued in 9 patients (18.4%) due to ILD.

Ten patients (20.4%) were diagnosed with abemaciclib-induced ILD. Details on ILD are shown in Table III. No significant differences were observed in the frequency of ILD between the left and right lungs. ILD mostly showed a pattern of organizing pneumonia (9 patients, 90%).

**Figure 2:**
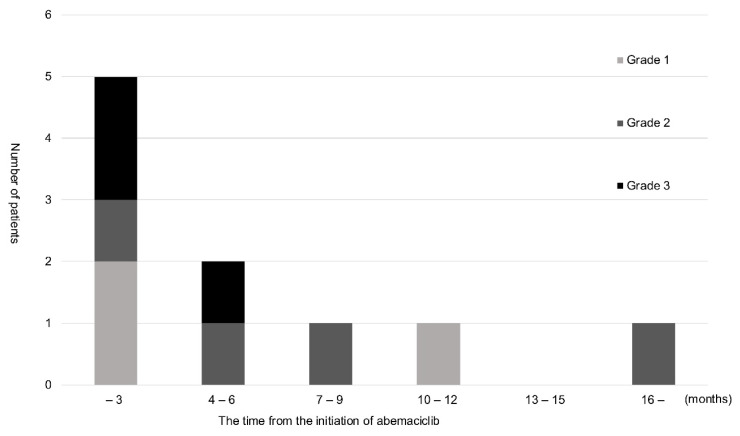
Time of onset and CTCAE grade of ILD The bar graph shows the time of onset of ILD. The vertical axis indicates the number of patients who developed ILD, and the horizontal axis shows the time from the initiation of abemaciclib. The darker the color of the bar graph, the higher the grade of interstitial pneumonia. The highest grade was 3. Ten patients developed ILD during the study period, with its onset occurring within 6 months of the initiation of abemaciclib in 7.

Figure 2 shows the severity of ILD based on CTCAE v5.0 and the timing of its onset. Seven of the 10 patients were diagnosed with ILD within 6 months of the initiation of abemaciclib, 3 of whom developed Grade 3 ILD during the same period. The remaining 4 patients developed Grade 1 and 2 ILD later and did not require treatment. Although there were no deaths from ILD, 4 patients were hospitalized and treated with steroids.

Table IV summarizes the results of univariate logistic regression analysis examining the association between each risk factor and the development of ILD. Age (≥65 vs. <65 years), history of radiotherapy, and later lines of treatment (≥3rd vs. 1st–2nd) were not significantly associated with ILD, with odds ratios of 0.70 (95% CI: 0.17– 2.80; p = 0.610), 0.27 (95% CI: 0.05–1.48; p = 0.132), and 0.56 (95% CI: 0.20–6.59; p = 0.881), respectively. In contrast, lung metastasis was significantly associated with ILD development (odds ratio = 5.00, 95% CI: 1.15– 21.70; p = 0.032), whereas bone and liver metastases were not. These results suggest that lung metastasis is a potential risk factor for ILD development.

## DISCUSSION

Drug-induced ILD is considered to result from multiple mechanisms. These include direct injury to alveolar epithelial or endothelial cells by the drug or its metabolites, as well as immune-mediated responses triggered by T cell activation. In some cases, oxidative stress and the release of proinflammatory cytokines may further contribute to pulmonary tissue damage (15). The development of ILD is a potential risk with many medications, and breast cancer treatments are no exception.

Recent advances have been achieved in drug therapy for ER-positive, HER2-negative breast cancer, particularly molecular-targeted drugs (13). While the development of molecular-targeted drugs has progressed, care is needed to prevent ILD (9, 14, 16). In phase III randomized studies showing the efficacy of abemaciclib, the incidence of ILD was very low at 2.3–3.4% (4, 6). On the other hand, the incidence of ILD was higher in Asian countries than in Western countries (10, 17, 18). For example, a single-center study from the National Cancer Center in Japan reported that the incidence of ILD was 13% (10).

We retrospectively analyzed the incidence of ILD induced by abemaciclib and its risk factors in our institution. The results obtained revealed a markedly higher frequency of abemaciclib-induced ILD than in other studies (4– 7, 10, 17, 18). We propose several possible reasons for this high rate of ILD. First, respiratory medicine specialists independently evaluated each CT image for ILD, which is the most essential feature and strength of this study. In previous studies, CT images were mostly evaluated by a single radiologist to assess the progression of breast cancer (4, 6, 10). Accurate assessments by respiratory specialists may have increased the sensitivity and specificity of the diagnosis of ILD. Second, ethnic background may be related to the incidence of ILD. All patients in the present study were Japanese, while most participants in phase III randomized studies were Caucasian. Previous studies indicated that ILD developed more frequently in Asians than in other races (10, 16, 17). Therefore, a genetic predisposition has been implicated and may have contributed to the high incidence of ILD in this study. Third, although age did not correlate with the incidence of ILD, the average age of patients was 65.7 (42–87) years, which was slightly older than in previous studies (4–7). Age has generally been associated with the development of drug toxicity, and an older age has been proposed as a risk factor for ILD (19). Finally, approximately 43% of patients treated with abemaciclib had a history of radiation therapy to the chest. A study on abemaciclib-induced ILD in Japan reported that 39% of patients had a history of radiation therapy (10), which was not significantly different from the present case. On the other hand, some reports indicate that the incidence of ILD did not increase even when CDK4/6 inhibitors, including abemaciclib, were used after radiation therapy (20, 21).

Of the cases that developed ILD, 70% were observed within the first 6 months of the administration of abemaciclib. This is consistent with previous findings showing that the onset of ILD was more common within 5 months of the initiation of abemaciclib (18). Although the timing of the onset of ILD remains controversial due to the small number of cases examined and the observation period, it is important to closely monitor ILD symptoms, including fever and cough, and evaluate interstitial shadows on CT soon after the initiation of abemaciclib. Grade 1 ILD was detected in 30% of patients in the present study, which was consistent with previous findings (4, 6). All patients with Grade 1 ILD remained asymptomatic with no subsequent progression to Grade 2 or higher.

Our research showed that the use of abemaciclib in patients with lung metastasis was more likely to cause severe ILD (odds ratio = 5.00, 95% CI: 1.15–21.70; p = 0.032). The interrelationship between these conditions and the mechanisms responsible has not yet been elucidated; however, some studies proposed underlying lung disease as a risk factor for ILD (22). Although a retrospective study reported that 60% of patients who developed CDK4/6 inhibitor-induced ILD had pulmonary metastases (23), the extent to which pulmonary metastases and abemaciclib contribute to the development of ILD remains unclear. The tumor microenvironment in metastatic lung lesion has been shown to support chronic inflammation and immune cell recruitment, including T lymphocytes and macrophages, which may contribute to local immune activation and increase susceptibility to drug-induced injury (24). In addition, in vitro studies have shown that CDK4/6 inhibitor treatment may induce cell cycle arrest and subsequent cellular senescence, which can lead to increased recruitment of inflammatory cells in the lung. This inflammatory infiltration has been suggested to play a central role in the pathogenesis of ILD (25, 26). Several patients required hospitalization and steroid pulse therapy; therefore, we need to pay more attention to patients with lung metastasis.

While this study has the strength, as noted above, of accurate CT evaluations by respiratory medicine specialists, there are some limitations. The decision to initiate and discontinue abemaciclib and the management of AEs mainly depend on each physician. In addition, the study included patients who used abemaciclib shortly after its launch and, thus, did not have a favorable management process for AEs. For example, approximately 30% of patients received abemaciclib as a 3rd-line treatment or later even though it is recommended as a 1st- or 2ndline treatment for metastatic or recurrent breast cancer (4–7). Although the number of treatment lines was not considered to be a risk factor for ILD in the multivariate analysis, this may have played a role in drug use and patient management. Furthermore, this was a single-center study with a small number of cases analyzed. Future studies on AEs are expected to be conducted on more patients at multiple centers. Larger multicenter studies in Asian populations are warranted to further clarify risk factors, including potential genetic predisposition, for abemaciclib-induced ILD.

## Figures and Tables

**Table I:** Patient characteristics (All = 49)

Age at the initiation of abemaciclib, mean (range)	65.7 (42–87)
Sex, n (%)	
Female	49 (100)
Male	0 (0)

Body mass index, mean (range)	22.9 (14.8–32.8)

Medical history, n (%)	
Asthma	9 (18.4)
COPD	1 (2.0)

Estrogen receptor, n (%)	
positive	49 (100)
negative	0 (0)
unknown	0 (0)

Progesterone receptor, n (%)	
positive	35 (71.4)
negative	14 (28.6)
unknown	0 (0)

Human epidermal growth factor receptor 2, n (%)	
positive	0 (0)
negative	48 (98.0)
unknown	1 (2.0)

History of smoking, n (%)	1 (2.0)

History of radiation therapy to the chest, n (%)	21 (42.9)

Baseline interstitial pneumonia, n (%)	0 (0)

**Table II:** Clinical characteristics (All = 49)

	n (%)
Combined endocrine therapy	
Aromatase inhibitor	13 (26.5)
Fulvestrant	35 (71.4)
Tamoxifen	1 (2.0)

Metastatic site (overlap)	
Bone	22 (44.9)
Liver	18 (36.7)
Lung	15 (30.6)

The treatment line	
1st	19 (38.8)
2nd	16 (32.7)
3rd	5 (10.2)
4th	3 (6.1)
5th or later	6 (12.2)

Previous chemotherapy for metastatic breast cancer, n (%)	13 (26.5)

Previous molecular target therapy, n (%)	
Palbociclib	6 (12.2)
Everolimus	2 (4.1)

**Table III:** Details of interstitial lung disease (n = 10)

	n (%)
CTCAE v5.0 Grade	
1	3 (30)
2	4 (40)
3	3 (30)

Site of ILD	
Right lung dominant	2 (20)
Left lung dominant	2 (20)
Bilateral lung	6 (60)

Pattern of pneumonia	
Organizing Pneumonia	9 (90)
Others	1 (10)

Treatment for ILD	4 (40)

**Table IV:** Results of univariate logistic regression analysis for ILD development

Variable	Univariate analysis		

	Odds Ratio	95% CI	p-value
Age (≥65 vs. <65)	0.70	0.17–2.80	0.610
BMI (≥25 vs. <25)	0.64	0.13–3.48	0.602
Asthma (Yes vs. No)	0.43	0.05–3.91	0.454
COPD (Yes vs. No)	NA	NA	NA
Radiation therapy (Yes vs. No)	0.27	0.05–1.48	0.132
Prior ILD (Yes vs. No)	NA	NA	NA
Bone metastasis (Yes vs. No)	0.78	0.19–3.20	0.727
Liver metastasis (Yes vs. No)	0.36	0.07–1.92	0.231
Lung metastasis (Yes vs. No)	5.00	1.15–21.70	0.032*
Line of treatment (≥3rd vs. 1st–2nd line)	0.56	0.20–6.59	0.881
Chemotherapy after recurrence (Yes vs. No)	0.25	0.10–3.06	0.505
Prior palbociclib treatment	NA	NA	NA
Prior everolimus treatment	NA	NA	NA

NA: Not applicable due to insufficient cases or lack of events for estimation.

* Statistically significant.
